# The Role of Antioxidants in Ameliorating Cyclophosphamide-Induced Cardiotoxicity

**DOI:** 10.1155/2020/4965171

**Published:** 2020-05-10

**Authors:** Muluken Altaye Ayza, Kaleab Alemayehu Zewdie, Bekalu Amare Tesfaye, Dawit Zewdu Wondafrash, Abera Hadgu Berhe

**Affiliations:** Department of Pharmacology and Toxicology, School of Pharmacy, Mekelle University, Mekelle, Ethiopia

## Abstract

The chemotherapeutic and immunosuppressive agent cyclophosphamide has previously been shown to induce complications within the setting of bone marrow transplantation. More recently, cardiotoxicity has been shown to be a dose-limiting factor during cyclophosphamide therapy, and cardiooncology is getting wider attention. Though mechanism of cyclophosphamide-induced cardiotoxicity is not completely understood, it is thought to encompass oxidative and nitrative stress. As such, this review focuses on antioxidants and their role in preventing or ameliorating cyclophosphamide-induced cardiotoxicity. It will give special emphasis to the cardioprotective effects of natural, plant-derived antioxidants that have garnered significant interest in recent times.

## 1. Introduction

### 1.1. Drug-Induced Cardiotoxicity

Drug-induced cardiotoxicity poses a serious risk to human health, and cardiooncology is currently becoming an important concern [1]. Antineoplastic treatments led to increased overall and progression-free survival in the management of an increasing number of malignancies [2]. However, as cancer survival has improved with advancing therapies, late cardiovascular adverse effects have become an important management issue, mainly in childhood cancers, leukaemia, lymphoma, and breast cancer. In patients diagnosed with early stage breast cancer, cardiovascular disease is the major cause of mortality [3]. Even though anticancer drugs are targeted against malignant cells, they are also toxic to normal cells [4].

Patients who survived cancer, when compared to their healthy counterparts, are at an increased risk of cardiovascular-related mortality, which might be due to myocardial infarction with coronary artery disease, cardiomyopathy with congestive heart failure, and cerebrovascular events [5, 6]. Patients on cancer chemotherapy can be considered as a stage A heart failure group, patients with increased risk of heart failure and do not have structural heart disease [7, 8]. Total dose of the anticancer agent patient received, rate of drug administration, extent of radiation of the mediastinum, age, being female, previous history of heart disease, and increased blood pressure are risk factors to develop cardiotoxicity [9, 10].

Antineoplastic agents are well known to cause a wide array of toxicities including cardiac dysfunction leading to heart failure, arrhythmias, myocardial ischemia, hypertension, thromboembolism, myocarditis, and pericarditis [11]. Anthracyclines are the best known of the chemotherapeutic agents that cause cardiotoxicity. In addition, alkylating drugs, including cisplatin, cyclophosphamide, ifosfamide, carmustine, chlormethine, busulfan, and mitomycin, are also linked with cardiac toxicity [9].

### 1.2. Cyclophosphamide

Cyclophosphamide is an alkylating, anticancer agent which was first characterized in experiments on rat tumors. It is an oxazaphosphorine-substituted nitrogen mustard, with strong cytotoxic and immunosuppressive activity [12]. It is the mainstay of most preparative regimens for organ transplant and a broadly active anticancer, immunosuppressive agent used in combination chemotherapy for Hodgkin's disease, non-Hodgkin's lymphoma, leukaemia, rheumatoid arthritis, Burkitt's lymphoma, lupus erythematosus, multiple sclerosis, neuroblastoma, multiple myeloma, endometrial cancer, breast cancer, and lung cancer. At high dosages, cyclophosphamide can be used alone or in combination with bone marrow transplant in the management of solid tumors and lymphomas [9, 13].

**Figure 1:**
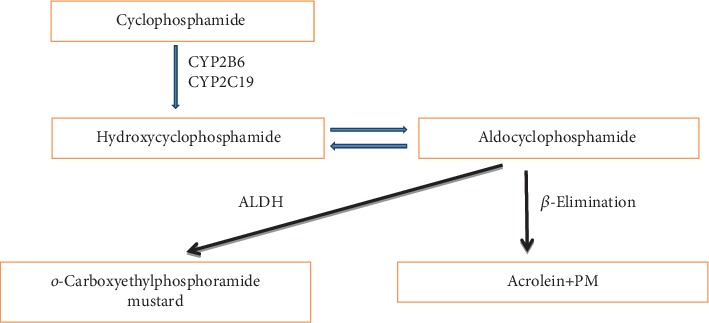
Major metabolic pathway of cyclophosphamide.

The electrophilic nature of the alkyl group enables the drug to react with nucleophilic moieties of DNA or proteins, and this leads to the covalent transfer of an alkyl group. Cyclophosphamide is a prodrug that requires an activation step by cytochromes (P450) in the liver [14]. As shown in Figure 1, the introduction of the hydroxyl group to the oxazaphosphorine ring generates 4-hydroxycyclophosphamide, which cooccurs in equilibrium with its isomer, aldophosphamide. Then, aldophosphamide is converted into two compounds, phosphoramide mustard and acrolein (Figure 1) [15].

Phosphoramide mustard forms a highly reactive cyclic aziridinium cation, which can react with the N(7) of the guanine and with cytidine from the DNA. Due to the two reactive moieties in the molecule, intrastrand and interstrand cross-links can be formed [16]. This leads to inhibition of DNA replication and apoptosis, with the active metabolites also having cell-cycle-independent activity. The specific mechanism of action of the compound used in managing autoimmune diseases has been postulated to include apoptosis, B-cell suppression, which will lead to decreased immunoglobulin G production and decreased production of adhesion molecules and cytokines [12].

Acrolein is the cause of hemorrhagic cystitis, one of the major toxicities of cyclophosphamide therapy. Other toxicities include bone marrow suppression, cardiotoxicity, gonadal toxicity, and carcinogenesis, with cumulative doses being the principal risk factor [15]. Additionally, administration of a single, large dose of cyclophosphamide is capable of causing hemorrhagic cell death, leading to heart failure or even death [17].

## 2. Pathophysiology of Cyclophosphamide-Induced Cardiotoxicity

Cyclophosphamide-induced cardiac damage is dose dependent, and the total dose of an individual course is the best indicator of toxicity, with patients who receive greater than 150 mg/kg or 1.55 g/m^2^/day, which are at a high risk for cardiotoxicity [18]. The dose-limiting factor during cyclophosphamide therapy is cardiotoxicity [19], which is irreversible [20]. Fatal cardiomyopathy has been reported among 2–17% of patients taking cyclophosphamide. It is dependent on the regimen and the particular patient population characteristics [21]. Overall, cyclophosphamide-induced cardiotoxicity affects between 7 and 28% of patients taking the drug [13].

The pathophysiology of cyclophosphamide-induced cardiac damage is poorly understood [10], although its metabolites are thought to induce oxidative stress and direct endothelial capillary damage with resultant extravasation of proteins, erythrocytes, and toxic metabolites. In the presence of toxic metabolites, breakdown of endothelial cells contributes to direct damage to the myocardium and capillary blood vessels resulting in edema, interstitial hemorrhage, and formation of microthrombosis [22, 23].

Endothelial cells are more susceptible to cyclophosphamide-induced damage than other cells ([24]); this might be associated with their high proliferation rate [25]; cyclophosphamide-induced reactive oxygen species generation can also lead to a reduction in nitric oxide bioavailability, thus leading to compromised endothelial function [26].

Molecular mechanisms of cyclophosphamide-mediated cardiac damage are currently being postulated, potentially leading to better preventative strategies to treat cardiotoxicity. It has been shown that treatments with cyclophosphamide inhibited heart-type fatty acid-binding proteins and carnitine palmitoyltransferase-I gene expression in cardiac tissues [27]. Inhibition of these pathways leads to decreased production of adenosine triphosphate and accumulation of toxic metabolites from fatty acid oxidation, consequently leading to cardiomyopathy [28]. Heart-type fatty acid-binding protein can be used as an early diagnostic marker of chemotherapy-induced cardiotoxicity [29]. In addition, carnitine deficiency can aggravate cardiotoxicity and it is important to monitor serum and urinary carnitine levels [30]. Carnitine supplementation showed beneficial effects in various cyclophosphamide-induced toxicities [31–34].

Cyclophosphamide administration affects the ability of the heart mitochondria to retain accumulated calcium [35]. Calcium leak from sarcoplasmic reticulum can lead to mitochondrial calcium overload, leading to reduced production of adenosine triphosphate and increased release of ROS [36]. It is reported that improving mitochondrial function through supplementation of lupeol and its ester can protect heart from cyclophosphamide-induced toxicity [37].

Cyclophosphamide is found to promote proinflammatory cytokines [38, 39]. It enhanced nuclear factor-kappa B (NF-*κ*B) phosphorylation, both expression and serum levels of cyclooxygenase-2 (COX-2), tumor necrosis factor-alpha (TNF-*α*), and interleukin-1 beta (IL-1*β*) [40, 41]. Nuclear erythroid 2-related factor 2 (Nrf2) and NF-*κ*B are considered as an important molecular target for the anti-inflammatory and antioxidant chemicals for cytoprotection during cyclophosphamide therapy [42, 43]. It has been reported that inhibition of the NF-*κ*B/TNF-*α* pathway prevented cyclophosphamide-induced multiple organ toxicity including the heart, kidney, and liver [44, 45].

It has been reported that p53 expression plays an important role in apoptosis [46, 47]. Reduction in apoptosis, infarct size, and hemodynamic parameter improvement can be achieved by inhibiting p53 [48]. Cyclophosphamide-induced activation of p53 protein is considered as one of the possible mechanisms for cardiomyopathy, and it is reported that probucol supplementation restored cyclophosphamide-induced upregulation of p53 and reversed apoptosis in cardiomyocytes [49].

**Figure 2:**
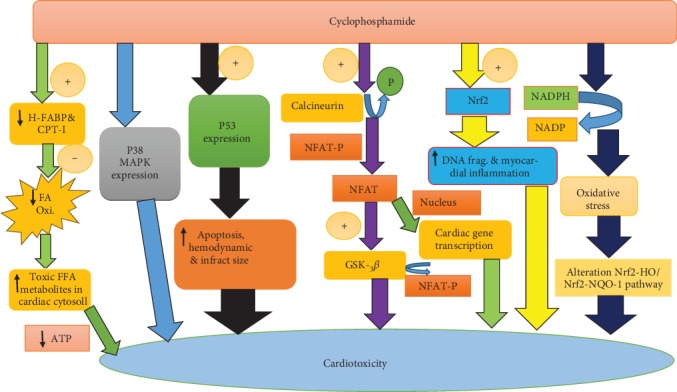
Molecular mechanisms involved in cyclophosphamide-induced cardiotoxicity.

Cyclophosphamide activates the p38 mitogen-activated protein kinase (p38-MAPK) pathway, and it can induce an oxidative injury [50]. Cyclophosphamide-enhanced proinflammatory/proapoptotic activities are reported to result in cardiomyopathy, myocardial infarction, and heart failure [13]. Rutin attenuated cyclophosphamide-induced oxidative stress and inflammation through downregulating TNF-*α*, IL-6, and expressions of p38-MAPK, NF-*κ*B, and COX-2 (Figure 2) [45].

Cyclophosphamide induces the calcineurin-mediated dephosphorylation of nuclear factor of activated T-cell (NFAT); it belongs to the family of calcium-regulated transcription factors. Unphosphorylated/active GSK-3*β* phosphorylates eIF2, NFAT, and c-jun and thus contributes significantly to cardiac hypertrophy inhibition/protection [13]. Cyclosporine A prevented NFAT nuclear translocation and reversed cyclophosphamide-induced cardiac damage [35, 51].

In general, mechanisms of cyclophosphamide-induced cardiotoxicity encompass oxidative and nitrative stress, protein adduct formation which leads to cardiomyocyte inflammation, altered calcium homeostasis, programmed cell death, swelling of the cardiomyocytes, nuclear splitting, vacuolization, and alteration in signaling pathways. These events result in diseases of the heart muscle including heart failure, if left undiagnosed or untreated, and may result in death [13]. Further supporting a role of cyclophosphamide-induced oxidative stress in the evident cardiotoxicity of the compound, exposure of rats to cyclophosphamide resulted in reduced pulmonary glutathione (GSH) content, GSH reductase (GRx), glucose-6-phosphate dehydrogenase, GSH peroxidase (GPx), and superoxide dismutase (SOD) activities [24].

## 3. Current Management for Cyclophosphamide-Induced Cardiotoxicity

Clinical management of cardiovascular diseases (CVDs) involves multiple drugs (angiotensin-converting enzyme inhibitors, blockers of angiotensin-II receptor, calcium channel blockers, *β*-blockers, aldosterone inhibitors, aspirin, statins, and warfarin), and others include diuretics, digoxin, and nitrates [52–54]. It is a common practice to use those medications in combination for the management of CVDs, and these lead to increased side effect and drug interactions [55].

These same preventive strategies can be considered for ischemia, heart failure, arrhythmia, hypertension, and arterial thromboembolism associated with cyclophosphamide-induced cardiotoxicity. Primary prevention may include widespread treatment of all patients who are potentially on cardiotoxic cancer treatments or early diagnosis of subclinical cardiac injury with targeted treatment.

According to the Canadian Cardiovascular Society recommendation, even though the recommendation is weak, patients believed to be at a high risk for cancer treatment-related left ventricular dysfunction, an angiotensin-converting enzyme inhibitor, angiotensin receptor blocker, and/or *β*-blocker, and/or statin can be considered to decrease the risk of cardiac damage [56]. Valsartan, an angiotensin receptor blocker, showed a strong effect in preventing acute cyclophosphamide-, doxorubicin-, vincristine-, and prednisolone-induced cardiotoxicity [57]. A nonselective *β*-blocker, carvedilol, with antioxidant activity and nebivolol, a selective *β*-blocker with a nitric oxide donor capacity, were reported to have an advantageous effect on antineoplastic-associated cardiac damage [58].

Mild to moderate heart failure and small pericardial effusions generally resolve within a few days to weeks after stoppage of cyclophosphamide. In the presence of suspected hemorrhagic myocarditis, cardiac tamponade, and cardiogenic shock, timely recognition and involvement of the intensive care unit or coronary care unit are vital. These patients need aggressive monitoring and circulatory support [22].

## 4. Natural Antioxidants for the Management of Cyclophosphamide-Induced Cardiotoxicity

The use of plants and plant-based products in the treatment of ailments has been known to mankind from ancient times [59]. Various natural antioxidants have originated from medicinal plants, which are used for the treatment of different ailments throughout the world, and there has been a significant interest in finding natural antioxidants from plant sources [60]. Diseases and drug-induced toxicities with the underlying cause of oxidative stress can be effectively managed with plants having antioxidant activity. Apart from being rich sources of antioxidants, phytochemicals are also known to impede the progression of cardiac tissue damage [59]. These compounds could serve as one of the valuable sources in industrial pharmaceutical research and can be treated as a complementary and alternative medicine.

Various medicinal plants showed cardioprotective activity against cyclophosphamide-induced cardiotoxicity in different preclinical studies (Table 1). In addition, xanthine-oxidase inhibitors (allopurinol and febuxostat) and nicorandil (vasodilatory drug used to treat angina) were also found to reverse cardiac damages induced by cyclophosphamide in male Wistar rats (Table 2).

## 5. Future Hopes and Hurdles Associated with Cardioprotective Antioxidants

Antioxidants such as flavonoids, flavones, isoflavones, anthocyanin, catechins, and isocatechins are the responsible ones for the antioxidant activity of spices and herb [90]. These led supplementation of antioxidants to be a popular practice to maintain optimal body function [91]. Polyphenols may reduce cholesterol absorption and upregulate hepatic mRNA abundance for the LDL receptor, reductions in plasma TG, yielding a reduced amount of LDL in circulation, and polyphenols were found to exert anti-inflammatory effects, thereby reducing the formation of cytokines involved in cellular adhesion [92]. The production of vasodilating factors like nitric oxide, endothelium-derived hyperpolarizing factor, and prostacyclin was enhanced by plant polyphenols. These plant phenols were also found to inhibit the production of vasoconstrictor endothelin-1 in endothelial cells and inhibit the expression of two main proangiogenic factors, matrix metalloproteinase-2, and vascular endothelial growth factor in smooth muscle cells [93]. Flavonoids can also improve endothelial function, and the primary mechanism for this is that the effect is nitric oxide production [94].

Even though the results were not posted, currently, there are different agents under clinical trial, including enalapril for prevention of chemotherapy-induced cardiotoxicity in high-risk patients (NCT00292526), nutritional supplement sulforaphane on doxorubicin-associated cardiac dysfunction (NCT03934905), estimation of the effects of ACE inhibitors and *β* blockers in the management of cardiotoxicity in oncologic patients (NCT02818517), cardiotoxicity prevention in breast cancer patients treated with anthracyclines and/or trastuzumab using bisoprolol and ramipril (NCT02236806), carvedilol effect in preventing chemotherapy-induced cardiotoxicity (NCT01724450), prevention of chemotherapy-induced cardiotoxicity in children with bone tumors and acute myeloid leukaemia using capoten (captopril) (NCT03389724), and statins to prevent the cardiotoxicity from anthracyclines (NCT02943590), and others are under investigation. These agents might be the future hopes for the management of chemotherapy-induced cardiotoxicity.

Even though antioxidants like flavonoids have a great hope in the future clinical scenario of cardioprotection [95], the importance of antioxidants is currently in question due to their less effectiveness in an *in vivo* study. These failures of antioxidants in preventing/treating diseases have become the main obstacle in the clinical scenario [96].

As concluded by Guallar et al., known antioxidants like vitamin E, *β*-carotene, vitamin A and B supplements, and folic acid are ineffective for the prevention of mortality and morbidity due to chronic diseases [97].

This failure might be due to different reasons including antioxidant-related reasons including testing incorrect antioxidant or combination of antioxidants; there might be differences between synthetic and dietary source antioxidants, reductive stress (i.e., too much antioxidant capacity), and it may also be related to patient or clinical trials [98].

Unconjugated flavonoid plasma level rarely exceeds 1 *μ*M, and metabolites of flavonoids have lower antioxidant activity. Since plasma total antioxidant capacities (TAC) are often in the range of 1 mM or more, it is difficult to picture how an additional 1 *μ*M polyphenol could exert an *in vivo* antioxidant effect. Antioxidants like flavonoids and other phenols are complex molecules and have multiple potential targets/actions in addition to antioxidant activity. These may include inhibition of different enzymes including cyclooxygenase, lipoxygenase, xanthine oxidase, matrix metalloproteinases, angiotensin-converting enzyme, proteasome, and cytochrome P450, affecting signal transduction pathways. Flavonoids may also interact with cellular drug transport systems [99]. These issues need to be addressed in the future.

## 6. Conclusion

Cyclophosphamide is a known anticancer and immunosuppressive agent that becomes effective after metabolic activation in the liver. Its wider clinical application is currently limited by its toxicity. Cardiotoxicity, which is associated with oxidative and nitrative stress, is one of the toxicities limiting the clinical use of cyclophosphamide. Different natural, plant-derived antioxidants (summarized in this review) showed significant cardioprotective effects in *in vivo* preclinical studies. However, further investigations aimed at improving their efficacy are required. Facilitating translational clinical research on those shown to be safe and effective in the preclinical studies should also be considered, lest the evidences from the preclinical studies would only be left to be discoursed in scientific meetings and publications.

## Figures and Tables

**Table 1:** Effect of medicinal plants and isolates against cyclophosphamide-induced cardiotoxicity.

References	Animals used	Method and intervention	Major findings
Asiri [49]	Male Wistar albino rats	Rats were administered with the same doses of corn oil (control) and probucol (61 mg/kg/day, i.p), respectively, for one week before and one week after a single dose of CP (200 mg/kg, i.p.).	Probucol prevented the development of CP-induced cardiotoxicity by a mechanism related, at least in part, to its ability to increase mRNA expression of antioxidant genes and to decrease apoptosis in cardiac tissues with the consequent improvement in mitochondrial oxidative phosphorylation and energy production.

Avci et al. [61]	Female Wistar albino rats	Animals were treated with 100 mg/kg/day silymarin (SLY) by oral gavage for 14 days and 30 mg/kg/day CP intraperitoneally starting from the seventh day and 100 mg/kg/day curcumin (CUR) by gavage for 14 days plus 30 mg/kg/day CP intraperitoneally starting from the seventh day.	Concurrent administration of SLY and CUR with CP resulted significantly lower biochemical parameters and histopathological and immunohistochemical results than in the CP-only group. It can be concluded that the natural antioxidant SLY and CUR might have protective effects against CP-induced cardiotoxicity and oxidative stress in rats.

Ayza et al. [62]	Either sex of Sprague Dawley rats	Animals were treated with a single dose of CP (200 mg/kg, i.p.) on the first day followed by hydromethanolic crude extract and solvent fractions of *Croton macrostachyus* for 10 days.	*Croton macrostachyus* reversed CP-induced elevations of cardiac troponin, ALT, AST, ALP, TC, and TG. These findings were further supported by histopathological findings.

Baniya et al. [63]	Male Wistar rats	Animals received a single dose of CP (200 mg/kg) on the first day followed by ethanolic extract of *Citrus grandis* (L.) Osbeck at 250 and 500 mg/kg, p.o. for 10 days.	Treatment with the extract reduced the serum biomarkers (CK-MB, ALT, AST, ALP, TC, and TG) and increased the tissue antioxidant level. Histopathology of heart tissue was also improved.

Bhatt et al. [64]	Either sex of Wistar rats	Cyclophosphamide (200 mg/kg, i.p.) toxicity was induced on day 1. Then, rats were treated with 100 mg/kg of mangiferin for 10 days.	Mangiferin treatment resulted in decrement of the serum cardiac biomarkers (AST, ALT, ALP, CK-MB, CK-NAC, and LDH). Mangiferin increased tissue antioxidant levels (SOD, CAT, and GSH), and animals showed improvement in lipid profile, ECG parameters, histological score, and mortality.

Bjelogrlic et al. [65]	Female BalbC/NIH mice	Animals were treated with vitamin E (100 IU/kg, orally) 24 hr before single bolus doses of doxorubicin (10 mg/kg, intravenously), or doxorubicin and CP (150 mg/kg, i.p.).	Vitamin E in a single oral dose failed to inhibit acute cardiotoxic activity of doxorubicin but suspended further progression of the heart muscle damage over the time. On the contrary, vitamin E did not attain cardioprotection against doxorubicin and CP in combination.

Cetik et al. [66]	Sprague-Dawley rats	Carvacrol administration was started three days before the CP application and continued till the end of experiment (six days).	Carvacrol at both the doses increased the GSH levels close to the control group GSH levels. Carvacrol at 5.0 and 10 mg/kg doses lowered the levels of serum ALT, AST, LDH, and CK-MB. Reduced inflammation and lipid peroxidation in the heart tissue and increase of serum GSH and total antioxidant capacity (TAS) levels were found when carvacrol was applied.

Chakraborty et al. [67]	Either sex Wistar albino rats	Rats were subjected to CP toxicity with the dose of (200 mg/kg i.p.) on day first. Then, treated with green tea extract (GTE) along with hydrochlorothiazide.	GTE dose dependently reduced CP-induced myocardial toxicity. Green tea when combined with hydrochlorothiazide reduced the associated side effects and exhibited myocardial protection.

Chakraborty et al. [68]	Male Wistar albino rats	Rats were treated with combination of curcumin (100, 50, 25 mg/kg, p.o.) and piperine (20 mg/kg, p.o.) for 10 days. All treated groups were subjected to CP (200 mg/kg, i.p.) toxicity on day 1.	Piperine incorporation with the doses of 50 and 25 mg/kg with curcumin exhibited a significant beneficial effect compared to the curcumin alone-treated group. Treatment with curcumin and piperine significantly modified the markers.

Conklin et al. [69]	Glutathione S-transferase (GSTP) wild-type (WT) and GSTP-P1/P2 null mice	To examine CP cardiotoxicity, WT and GSTP-null mice were treated with saline (control) or 100, 200, and 300 mg/kg CP.	According to the findings, myocardial GSTP levels are likely to be key determinants of CP cardiotoxicity. GSTP is a highly regulated enzyme that is readily induced by different environmental factors, diet constituents such as garlic organosulfur compounds, coffee, and chemopreventive agents such as selenocysteine conjugates. Therefore, GSTP induction by such agents could attenuate CP toxicity, and conversely, disturbed metabolic states such as obesity, which are associated with downregulation of GSTP, and could enhance the cardiotoxicity of CP treatment.

El-Agamy et al. [70]	Male Wistar rats	Cardiotoxicity was induced by single injection of CP (200 mg/kg, i.p.). Methyl palmitate (MP) was administered at two different dose levels (300 and 400 mg/kg) for 10 days before and 7 days after CP injection.	Animals treated with MP showed significant attenuation of ECG changes. MP supplementation significantly lowered the elevated cardiac markers and improved cardiac lesions, which was more prominent at the higher dose. MP treatment significantly decreased MDA content and enhanced the antioxidant parameters (SOD and GSH), and it significantly decreased the expression of TLR4 and NF-*κ*B p65. MP supplementation suppressed inflammatory cytokines (TNF-*α* and nitrite/nitrate) and reduced apoptosis.

Gado et al. [71]	Male Swiss albino rats	Curcumin (200 mg/kg, i.p.) was administered for 8 consecutive days followed by a single dose of CP (150 mg/kg, i.p.).	Serum LDH and CPK were decreased significantly with the curcumin administration. Curcumin treatment significantly decreased MDA, NO(x), and restore GSH level in the cardiac tissue. Histological alterations were also found to be improved.

Gunes et al. [72]	Male Sprague Dawley rats	Animals received respective selenium (Se) doses (0.5 or 1 mg/kg) for 6 days and then a single dose of CP administered on the sixth day. On day 7, the animals were sacrificed.	Based on microscopic evaluation, tissue damage was noticeably lower in CP plus Se groups. Additionally, 1 mg/kg Se was more protective than 0.5 mg/kg Se. It can be concluded that Se can be a potential candidate to ameliorate CP-induced cardiotoxicity which may be related to its antioxidant activity.

Iqubal et al. [73]	Male Swiss albino mice	Animals were treated with nerolidol (NER) (200 and 400 mg/kg p.o.) and fenofibrate (FF) 80 mg/kg, p.o. for 14 days along with a single dose of CP 200 mg/kg i.p. on the 7th day.	NER 400 significantly reversed cardiotoxic effects of CP and showed cardioprotective activity which was comparable with FF 80. However, NER 200 did not show significant cardioprotective activity.

Mansour and Hasan [74]	Male Wistar albino rats	Rats were pretreated with N-acetylcysteine (200 mg/kg) for 5 days; 1 hour after the last dose, rats were injected with CP (200 mg/kg).	Treatment with N-acetylcysteine significantly decreased serum levels of ALT, AST, CK, and LDH. Decrease in the NOx, MDA levels and TNF- *α*, SOD, catalase, GSHPx, and GST levels were increased.

Mythili et al. [75]	Male Wistar albino rats	Rats received single injection of CP (200 mg/kg, i.p.) to induce cardiotoxicity, then followed by dl-*α*-lipoic acid treatment (25 mg/kg for 10 days).	Normalized lipid peroxidation and antioxidant defenses were observed in the dl-*α*-lipoic acid-treated rats.

Mythili et al. [76]	Male Wistar albino rats	Rats were injected with a single dose of CP (200 mg/kg, i.p) to induce cardiotoxicity, and then rats were treated with lipoic acid (25 mg/kg, orally for 10 days).	Treatment with lipoic acid reversed the abnormalities in the lipid levels and activities of lipid-metabolizing enzymes to near normalcy.

Mythili et al. [77]	Male Wistar albino rats	Rats received CP (200 mg/kg i.p.), which is immediately followed by lipoic acid (25 mg/kg orally) for 10 days.	Lipoic acid effectively reversed abnormal biochemical changes to near normalcy. Based on the results, lipoic acid showed a protective role of lipoic acid in CP-induced cardiotoxicity.

Nagi et al. [78]	Male Wistar albino rats	Rats received thymoquinone (50 mg/l in drinking water) for 5 days before a single dose of CP (200 mg/kg, i.p.) and continued thereafter until day 12. On day 13, animals were sacrificed.	Thymoquinone reversed CP-induced increase in serum CK-MB and LDH. Complete reversal of the CP-induced increase in serum cholesterol, triglycerides, urea, and creatinine to the control values. CP-induced increase in TBARS and NO(x) and a decrease in GSH, GPx, and CAT were reversed by thymoquinone supplementation. Thymoquinone supplementation to CP-treated rats completely reversed the increase in TNF-*α* induced by CP.

Omole et al. [79]	Male Wistar rats	Rats were pretreated with 200 and 400 mg/kg/d Kolavorin, orally for 14 days followed by CP (50 mg/kg/d, i.p.) for 3 days.	Kolavorin pretreatment increased food consumption, body weight, and attenuated the biochemical and histological changes. It was reported that kolavorin inhibited oxidative stress and preserved the activity of antioxidant enzymes.

Sekeroğlu et al. [80]	Male Swiss albino mice	After treatment with *Viscum album* and quercetin for 7 days, rats were administered CP (40 mg/kg, i.p) on days 8 and 9 of the experiment. Total treatment period was 10 days.	Treatments decreased the levels of antioxidant enzymes, glutathione-S-transferases; reduced glutathione and mitotic index were observed. Quercetin completely and *Viscum album* partly ameliorated almost all of the examined parameters when given together with CP.

Senthilkumar et al. [81]	Male albino Wistar rats	Animals were cotreated with CP intraperitoneally dissolved in saline, in a dose of 150 mg/kg b.w. and different doses of squalene for the first 2 days, and squalene treatment was followed continuously, daily for 10 days up to the end of the experimental period.	Squalene oral treatment exerted protection to the heart, kidney, and liver at a dose of 0.4 ml/day/rat. Histopathological examinations also confirmed the protective efficacy of squalene. It can be concluded that squalene may be efficacious as a cytoprotectant in CP-induced toxicities.

Shalaby et al. [82]	Male Sprague-Dawley albino rats	Rats received *Zingiber officinale* 200 mg/kg/day orally followed by a single dose of CP (150 mg/kg i.p.).	Results showed significant improvement in the *Zingiber officinale*-treated group. Based on their conclusion, the cardiotoxic effect of CP might be prevented by *Zingiber officinale* supplementation.

Shanmugarajan et al. [83]	Male Wistar rats	Rats were treated with the methanolic leaf extract of *Ficus hispida* Linn. for 10 consecutive days following CP-induced oxidative myocardial injury on the first day.	Treatment with *Ficus hispida* Linn. decreased serum cardiac biomarkers (CPK, LDH, AST, and ALT), and these were increased in the heart tissue. *Ficus hispida* Linn. increased the levels of enzymic antioxidants (SOD, CAT, GPx, GSH, and GRx).

Song et al. [84]	Male ICR mice	Animals were injected with a single dose of CP (200 mg/kg i.p.) followed by the intragastric treatment with ferulic acid (FA) (50, 100 mg/kg) for 7 consecutive days.	FA significantly decreased the serum levels of cardiac biomarkers, IL-6, IL-1*β*, and TNF-*α* in CP-injected mice. Additionally, FA effectively reduced the total numbers of WBCs, RBCs, platelets, and hemoglobin content. FA also attenuated the histological changes of the heart tissues caused by CP. Moreover, western blot demonstrated that FA inhibited the phosphorylations of the NF-*κ*B signaling pathway in CP-stimulated cardiac tissues.

Sudharsan et al. [85]	Male Wistar albino rats	Rats were injected with a single dose of CP (200 mg/kg, i.p) and treated with lupeol and lupeol linoleate (50 mg/kg).	Lupeol and its ester reversed alterations of serum lipoproteins and lipid fractions in both serum and cardiac tissue. It was found that lupeol linoleate was more effective than lupeol.

Swamy et al. [86]	Male Wistar albino rats	Cardiotoxicity was induced by administering CP (200 mg/kg, i.p.) single injection. *Saraca indica* (200 and 400 mg/kg, p.o.) was daily for 10 days.	Treatment with *Saraca indica* reversed the status of cardiac biomarkers (CK, CK-MB, LDH, AST, ALT, and ALP) ECG, oxidative enzymes (GSH, SOD, and CAT), and lipid profile.

Todorova et al. [87]	Male Fischer 344 rats	After 2 d of prefeeding with glutamine (GLN) or glycine (GLY) by gavage, the rats were randomized into one of six groups receiving a lethal intraperitoneal dose of CP (450 mg/kg), a sublethal dose of CP (200 mg/kg), or saline (control).	The results showed that dietary GLN decreased cardiac necrosis and maintained normal cardiac GSH levels. GLN protected against the acute cardiotoxic effects of CP and significantly improved the short-term survival after lethal and sublethal doses of CP.

**Table 2:** The effect of currently available drugs against cyclophosphamide-induced cardiotoxicity.

References	Animals used	Methods and intervention	Main findings
El-Sheikh et al. [88]	Male Wistar rats	Rats were treated with allopurinol (ALL) 100 mg/kg/day or febuxostat (FEB) 10 mg/kg/day which were administered orally to rats in the presence and absence of CP (200 mg/kg i.p. single dose at the ninth day) treatment.	Based on the results, both xanthine oxidase (XO) inhibitors, ALL and FEB, ameliorated CP-induced cardiotoxicity. Though only FEB showed protective activity against CP-induced myelotoxicity, however ALL might aggravate myelotoxicity. Based on the findings, ROS and XO enzymatic pathways may largely participate in the mechanism of pathogenesis of cardiac and bone marrow toxicities related to CP exposure.
Refaie et al. [89]	Male Wistar albino rats	Rats were administered with nicorandil (NIC) (3 mg/kg/day) alone and coadministered with nitro-*ω*-l-arginine (L-NNA) (25 mg/kg/day) and glibenclamide (GP) (5 mg/kg/day) orally for 5 days and injected with CP (150 mg/kg/day) i.p. on 4^th^ and 5^th^ days.	NIC reversed CP-induced cardiotoxicity by its potassium channel opening effect, stimulating eNOS gene expression, anti-inflammatory, antiapoptotic, and antioxidant properties. Cotreatment with GP or L-NNA decreased the protective effect of NIC.

## Abbreviations

CAT: Catalase
CP: Cyclophosphamide
CVDs: Cardiovascular diseases
GPx: Glutathione peroxidase
GRx: Glutathione reductase
GSH: Glutathione.
